# An ultrasound model for stress urinary incontinence at six weeks postpartum

**DOI:** 10.1515/med-2026-1549

**Published:** 2026-10-30

**Authors:** Xia Zhang, Yang Long, Shan Liu, Limin Liu, Fenghuang Zhou, Wenjie Liu, Bing Zhou

**Affiliations:** Department of Medical Ultrasonics, The Second Affiliated Hospital, University of South China, Hengyang, Hunan, China; Department of Anaesthesiology, The Second Affiliated Hospital, University of South China, Hengyang, Hunan, China; Department of Emergency, The Second Affiliated Hospital, University of South China, Hengyang, Hunan, China; Department of Obstetrics, The Second Affiliated Hospital, University of South China, Hengyang, Hunan, China

**Keywords:** urinary incontinence, stress, postpartum period, pelvic floor, ultrasonography, nomograms

## Abstract

**Objectives:**

Stress urinary incontinence (SUI) after childbirth has both clinical risk factors and measurable changes in urethral support. Whether integrating these complementary features can identify affected women at six weeks postpartum remains uncertain.

**Methods:**

This retrospective, single-centre study included 454 women, divided chronologically into development (n=351) and temporal internal-validation (n=103) cohorts. LASSO selected ultrasound predictors. Ultrasound-only and combined clinical–ultrasound models were evaluated for discrimination, calibration and clinical utility.

**Results:**

SUI prevalence was 17.7 % and 26.2 % in the development and validation cohorts, respectively. LASSO retained bladder-neck mobility (BNM) and urethral funneling; the combined model additionally included age, vaginal delivery and overweight. The combined model outperformed the ultrasound-only model in development (C-statistic, 0.780 [95 % CI, 0.717–0.843] vs. 0.703 [0.634–0.771]; p=0.002). In validation, the corresponding C-statistics were 0.805 (0.716–0.894) and 0.737 (0.633–0.841), but the difference was not significant (p=0.141). The validation calibration intercept, slope and Brier score were 0.704, 1.054 and 0.161, respectively. Decision-curve analysis indicated potential net benefit at selected thresholds; continuous-predictor analysis yielded similar discrimination.

**Conclusions:**

Combining clinical and transperineal ultrasound features improved concurrent SUI identification over ultrasound features alone. External validation is required before clinical implementation.

## Introduction

Stress urinary incontinence (SUI) is the complaint of involuntary loss of urine on effort or physical exertion, or on sneezing or coughing, and is the most common form of female urinary incontinence [1], 2]. Pregnancy and vaginal childbirth are major risk factors, because stretching, compression and denervation of the pelvic floor during delivery weaken urethral support; consequently, SUI is highly prevalent in the postpartum period and frequently persists or worsens at middle age [3], [4], [5], [6]. Postpartum SUI is substantially under-diagnosed: many women do not report symptoms spontaneously, and untreated symptoms are associated with anxiety, depression, impaired quality of life and avoidance of physical and social activity [4], 7].

In keeping with current ICS terminology, uncomplicated SUI is primarily a symptom-based diagnosis established from characteristic symptoms and clinical evaluation; routine urodynamic testing is not required before initial conservative management and does not improve outcomes in uncomplicated cases [8]. Early identification matters because conservative therapy – above all supervised pelvic-floor muscle training (PFMT), physiotherapy, behavioural and lifestyle interventions, and structured postpartum rehabilitation – is most effective when started early [9], [10], [11], [12]. A practical tool that flags affected women at the routine six-week postpartum visit would therefore be clinically useful, provided it is explicit that such a tool identifies women who are already symptomatic at that visit rather than predicting who will develop SUI later.

Transperineal (translabial) pelvic-floor ultrasound is a non-invasive, reproducible modality that dynamically visualises the bladder neck, urethra and levator hiatus at rest and during the Valsalva manoeuvre [13]. Bladder-neck mobility (BNM, the craniocaudal displacement of the bladder neck between rest and maximal Valsalva), bladder-neck descent, the retrovesical angle, the urethral inclination and rotation angles, and internal urethral orifice (bladder-neck) funneling have all been related to impaired urethral support and SUI [14], [15], [16], [17], [18], [19], [20]. Importantly, bladder-neck motion can also be imaged with simpler, widely available transabdominal ultrasound, which may be better suited to primary-care screening and repeated follow-up [21], [22], [23]; the present study instead uses 4D transperineal ultrasound, which captures the full set of anatomical parameters.

Several prediction models for female or postpartum SUI have been published, most based only on questionnaires and obstetric history [24], [25], [26], and machine-learning approaches in this field are expanding rapidly [26]. Chen et al. combined pelvic-floor ultrasound with clinical factors during pregnancy [24], but models built specifically at the six-week postpartum visit remain scarce, and the incremental value of different variable blocks has seldom been tested formally. We therefore (i) used LASSO to screen twelve transperineal ultrasound parameters, (ii) built an ultrasound-only model (Model 1) and a combined clinical–ultrasound model (Model 2), and (iii) compared their discrimination, calibration and clinical net benefit in a temporally separated internal-validation cohort. This design quantifies the gain from adding clinical characteristics to ultrasound; because no clinical-only comparator was fitted, the incremental value of ultrasound over clinical factors alone cannot be estimated directly and is examined in the Limitations. Because every predictor and the SUI outcome were captured at the same visit, we frame the work as identification (case-finding) of concurrent SUI, reported according to TRIPOD [27].

## Materials and methods

### Study design, setting and participants

This retrospective, single-centre study was conducted at the Second Affiliated Hospital, Hengyang Medical School, University of South China. We reviewed all women who attended the routine six-week postpartum visit and underwent pelvic-floor ultrasound between January 2020 and December 2021 (n=501). Inclusion criteria were: (1) six-week postpartum review; (2) completion of the symptom-screening questionnaire (and the ICIQ-SF when the screen was positive); and (3) a complete, technically adequate transperineal ultrasound examination at rest and during Valsalva. Women with a history of perineal trauma or pelvic-floor surgery, urinary symptoms before pregnancy, inability to perform an adequate Valsalva manoeuvre, or a current urinary-tract infection were not eligible. Among the 501 women who underwent ultrasound, 15 declined the questionnaire and 32 had incomplete key clinical or ultrasound data, leaving 454 participants for analysis (Figure 1). By examination date, the first 351 women (January 2020–June 2021) formed the development cohort and the subsequent 103 (July–December 2021) formed the temporal internal-validation (test) cohort. This temporal split from a single centre constitutes internal rather than external validation. The study was reported in accordance with TRIPOD [27].

**Figure 1:**
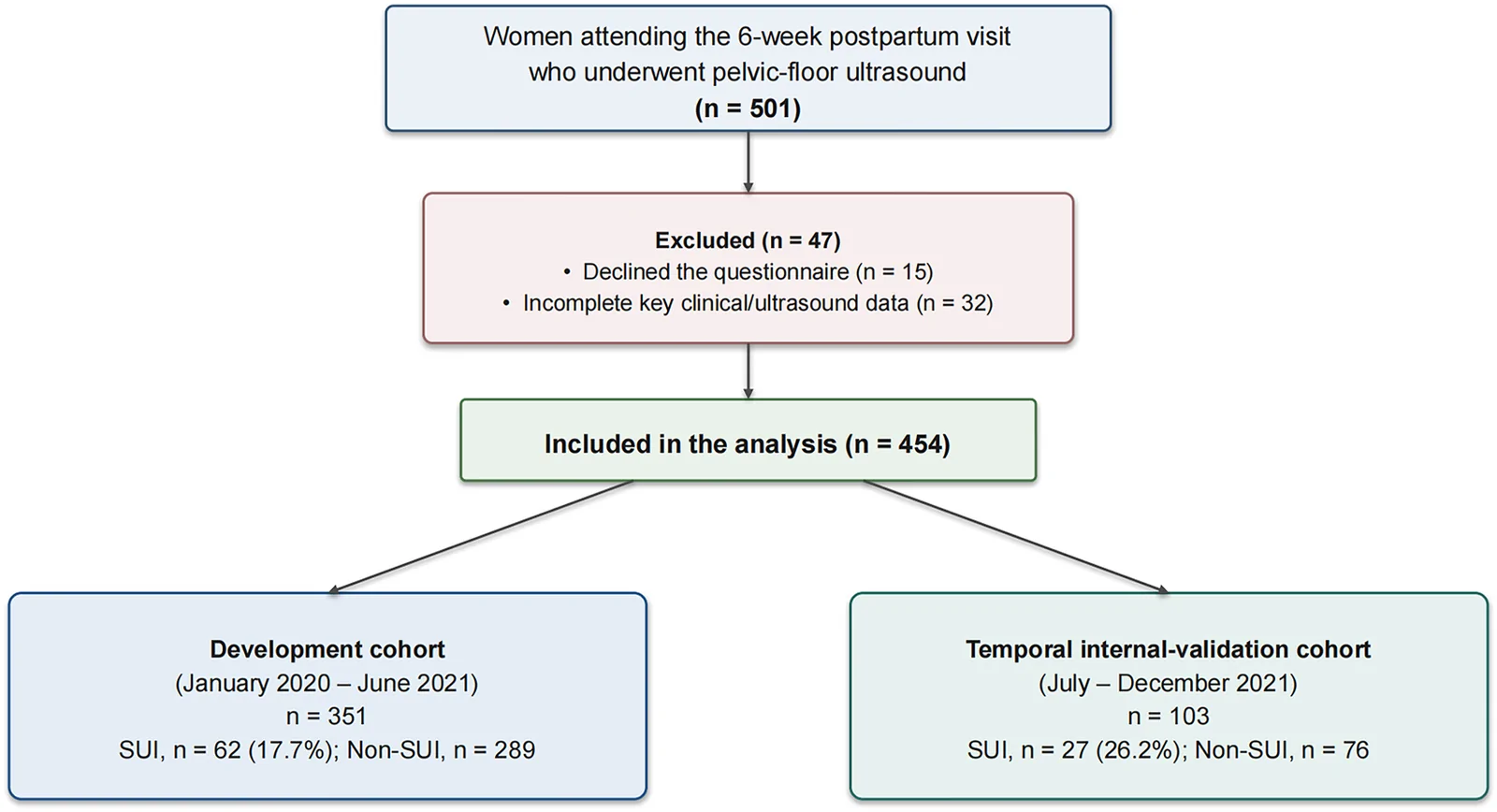
Patient flow diagram. Of 501 women attending the six-week postpartum visit who underwent pelvic-floor ultrasound, 454 were included and divided by examination date into a development cohort and a temporal internal-validation cohort.

The study was conducted in accordance with the Declaration of Helsinki and was approved by the Institutional Review Board of the Second Affiliated Hospital of University of South China (fast-track approval No. 2026043). Owing to the retrospective, de-identified design, the requirement for written informed consent was waived by the Institutional Review Board.

### Ascertainment of SUI and clinical variables

SUI was ascertained by a two-step symptom interview administered by a trained research staff member who was separate from the ultrasound operator. Women first answered a single screening question on leakage during coughing, sneezing, laughing, lifting or exercise; those responding affirmatively then completed the International Consultation on Incontinence Questionnaire–Short Form (ICIQ-SF) [28]. Consistent with ICS symptom-based terminology [1], 2], a woman was classified as having SUI when she reported involuntary leakage specifically associated with increased intra-abdominal pressure; no minimum ICIQ-SF score was required for SUI classification, and the ICIQ-SF was used to characterise symptom frequency and impact. A cough stress test, pad test and urodynamic testing were not performed; this is acknowledged as a possible source of misclassification in the Limitations. Clinical variables comprised maternal age, parity (primiparous/multiparous), mode of delivery, BMI measured at the six-week visit concurrently with ultrasound, and neonatal birth weight. Following the Chinese overweight/obesity classification, BMI >24 kg/m^2^ was defined as overweight (and BMI ≥28 kg/m^2^ as obesity); only the overweight threshold is modelled here. Women not meeting these SUI criteria formed the non-SUI group (women without identified SUI); this label does not assert the absence of urge or mixed incontinence.

### Transperineal ultrasound protocol

All examinations were performed by one experienced sonographer (eight years of dedicated pelvic-floor ultrasound experience) using an ultrasound system (GE Voluson E8, USA) equipped with a four-dimensional abdominal probe (4.0–8.0 MHz). The operator was blinded to the questionnaire results because symptom data were collected independently by a separate research staff member. Women were examined after emptying the bladder, in the dorsal lithotomy position, with the covered transducer placed on the perineum. After a resting acquisition, each woman performed the Valsalva manoeuvre for at least 6 s, repeated three times; measurements were taken from the frame of maximal Valsalva. With the inferoposterior margin of the pubic symphysis as the fixed reference, twelve ultrasound parameters were derived (Figure 2): at rest, bladder-neck distance (BNd; distance from the symphyseal reference to the bladder neck), levator ani hiatus area (LAGAR), urethral tilted angle (UTa) and posterior vesicourethral angle (PVa); at maximal Valsalva, BNd, bladder distance (distance from the symphyseal reference to the most dependent point of the posterior bladder wall, which differs from BNd because BNd references the bladder neck), LAGAR, UTa, PVa and urethral rotation angle (URa); and, as derived/dynamic measures, bladder-neck mobility (BNM; craniocaudal displacement of the bladder neck between rest and maximal Valsalva, in mm) and internal urethral orifice funneling (present/absent). A formal intraobserver/interobserver reliability sub-study was not performed and is acknowledged as a limitation.

**Figure 2:**
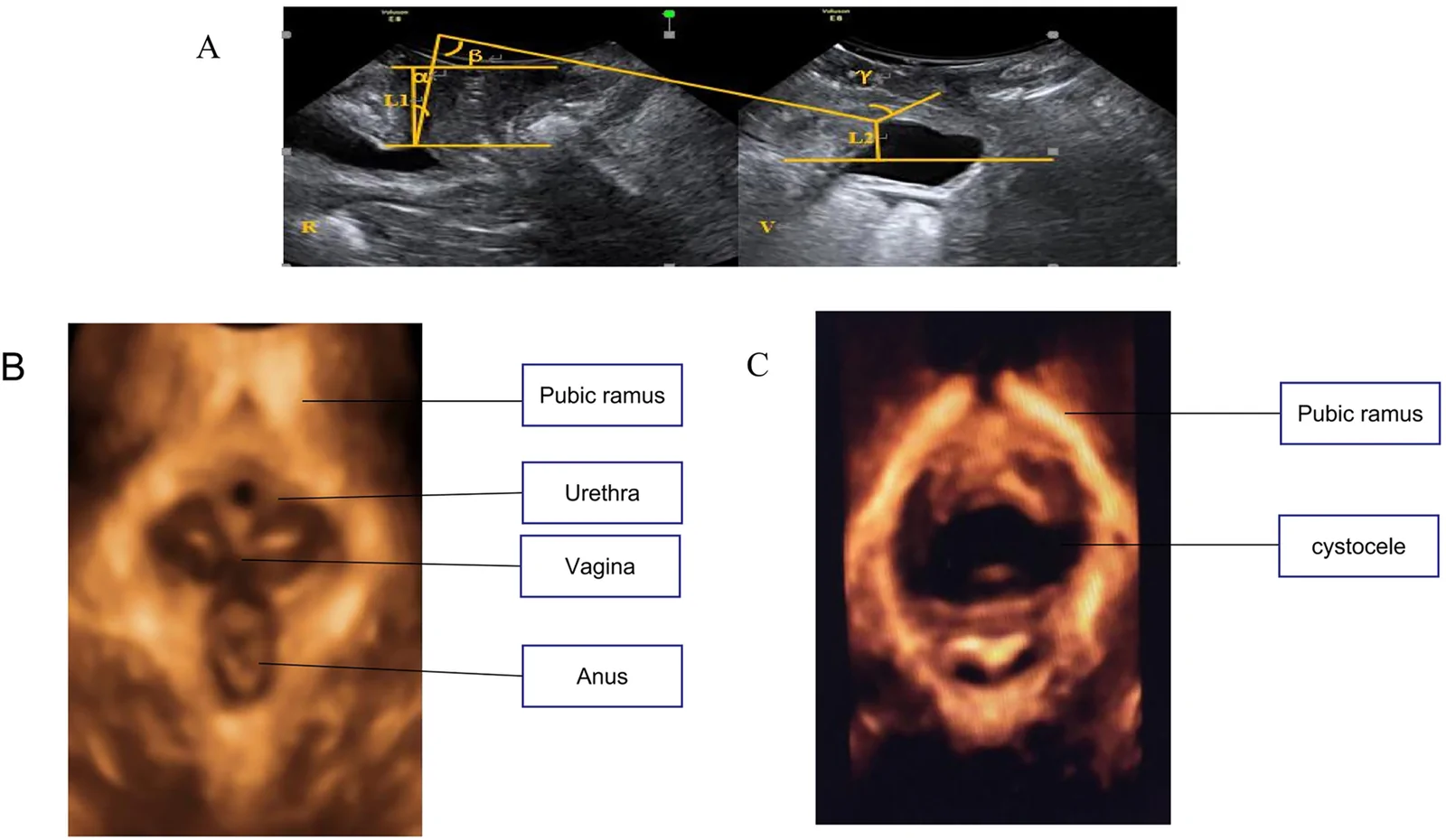
Transperineal pelvic-floor ultrasound measurements. (A) Two-dimensional sagittal imaging at rest (R) and at maximal Valsalva (V). L1 and L2 are the bladder-neck distances at rest and during Valsalva, respectively, and bladder-neck mobility (BNM)=L1 − L2; α is the urethral tilt angle (UTa), β the urethral rotation angle (URa) and γ the posterior vesicourethral angle (PVa). (B) Four-dimensional axial view at rest showing the pubic ramus, urethra, vagina and anus. (C) Four-dimensional axial view during Valsalva showing a cystocele.

### Predictor selection and model development

Analyses used complete records; no imputation was required in the final dataset because participants with missing key variables were excluded (Figure 1). Continuous ultrasound variables were standardised internally (z-scores) before penalised regression, whereas the binary funneling indicator was entered untransformed; the penalised coefficients shown are on the original variable scale. In the development cohort the twelve ultrasound parameters were screened by LASSO logistic regression with ten-fold cross-validation; the penalty was set at one standard error of the minimum (lambda.1se=0.0733; log λ=−2.61) to favour a parsimonious model [29]. Clinical candidate variables were first assessed by univariable logistic regression and then entered into a multivariable logistic regression; variables retained on clinical and statistical grounds were age, vaginal delivery and overweight. BNM was dichotomised at the Youden-optimal cut-off derived solely from the development cohort (34.5 mm). Two models were then fitted: Model 1 (ultrasound only: elevated BNM plus funneling) and Model 2 (combined: age, vaginal delivery, overweight, elevated BNM and funneling). The final Model 2 contained five predictors for 62 development-cohort events (a descriptive events-per-variable ratio of 12.4). Because this ratio uses only the final predictors and ignores the larger number of candidate ultrasound and clinical variables screened during selection, it should not be taken as proof of adequate sample size, and some overfitting risk remains [30]. Model 2 was presented as a nomogram to obtain individual probabilities.

### Performance assessment and validation

Discrimination was quantified by the concordance (C-) statistic/area under the ROC curve (AUC) with 95 % CIs derived by the DeLong method [31]; the paired DeLong test was used to compare Model 1 and Model 2 within the same cohort. In the development cohort we additionally report the bootstrap optimism-corrected C-statistic (2000 resamples). Calibration was assessed with calibration plots together with the calibration intercept and slope, the Brier score (lower is better; 0.25 corresponds to chance at 50 % prevalence) and the Hosmer–Lemeshow test [32]; calibration was not judged from the Hosmer–Lemeshow test alone. Clinical value was evaluated by decision-curve analysis [33], calculated separately in the test cohort (and in the development cohort for completeness), comparing each model with the treat-all and treat-none strategies across threshold probabilities. To address the information loss from dichotomising continuous variables, a sensitivity analysis refit Model 2 with BMI and BNM retained as continuous predictors.

### Statistical software

Analyses were performed in R 4.4.2 (R Foundation, Vienna, Austria), using the glmnet package for LASSO, pROC for ROC curves and DeLong comparisons, rms for calibration and nomograms, and rmda for decision-curve analysis. Continuous variables are shown as mean (standard deviation) when approximately normally distributed or median [interquartile range] otherwise; groups were compared with Welch’s independent-samples t-test for approximately normal continuous variables, the Wilcoxon rank-sum test for skewed continuous variables, and the Pearson chi-square test (or Fisher’s exact test when any expected cell count was below five) for categorical variables, and the corresponding statistic (t, Z or χ^2^) is reported. Two-sided p<0.05 was considered significant.

## Results

### Participants and cohort comparison

Of 501 women assessed for eligibility, 15 declined the questionnaire and 32 had incomplete key clinical or ultrasound data; 454 women were analysed (Figure 1). The development cohort comprised 351 women, of whom 62 (17.7 %) had SUI; the temporal test cohort comprised 103 women, of whom 27 (26.2 %) had SUI (between-cohort difference in prevalence, Pearson χ^2^ p=0.055). The two cohorts were broadly comparable: of all characteristics examined, only the proportion of vaginal delivery (67.2 % vs. 53.4 %, p=0.010) and resting levator hiatus area (median 14 vs. 13 cm^2^, p=0.049) differed between cohorts (Supplementary Table S1).

### Baseline characteristics

Characteristics of the non-SUI and SUI groups within each cohort are shown in Table 1. In the development cohort, women with SUI were older, were more often multiparous and vaginally delivered, were more often overweight, and showed greater bladder-neck descent, larger Valsalva hiatus and urethral angles, greater BNM and more frequent funneling (all p<0.05). The same direction of differences was observed in the smaller test cohort.

**Table 1:** Baseline characteristics of the non-SUI and SUI groups in the development and temporal test cohorts.

Characteristic	Non-SUI (n=289)	SUI (n=62)	Z/χ^2^/t	p-Value	Non-SUI (n=76)	SUI (n=27)	Z/χ^2^/t	p-Value
	Development				Temporal test			
Age, years	28.0 [26.0, 31.0]	30.0 [27.3, 32.0]	2.89	0.004	29.0 [26.0, 31.0]	30.0 [27.0, 34.0]	1.18	0.238
Multiparity	94 (32.5)	31 (50.0)	6.80	0.009	25 (32.9)	13 (48.1)	1.99	0.158
Vaginal delivery	183 (63.3)	53 (85.5)	11.38	0.001	35 (46.1)	20 (74.1)	6.29	0.012
BMI at 6-week visit, kg/m^2^	22.27 [20.06, 24.22]	22.58 [20.70, 24.34]	1.41	0.159	21.99 [20.25, 24.26]	24.23 [21.81, 24.75]	2.23	0.026
Overweight (BMI>24)	77 (26.6)	28 (45.2)	8.35	0.004	23 (30.3)	16 (59.3)	7.12	0.008
Birth weight, kg	3.30 [3.00, 3.60]	3.33 [3.10, 3.60]	0.51	0.612	3.25 [3.05, 3.50]	3.23 [3.10, 3.40]	0.08	0.934
**At rest**								
BNd, mm	29.09 (3.15)	29.24 (3.50)	0.32	0.753	28.88 (2.85)	28.37 (2.88)	−0.79	0.431
LAGAR, cm^2^	14.0 [12.0, 16.0]	14.0 [13.0, 16.0]	1.55	0.121	13.0 [11.0, 14.0]	14.0 [13.0, 16.0]	2.63	0.009
UTa (°)	24.0 [11.0, 37.0]	21.5 [9.3, 32.5]	1.45	0.148	27.0 [14.0, 38.3]	18.0 [12.5, 37.5]	1.01	0.313
PVa (°)	115.0 [103.0, 127.0]	117.0 [105.0, 127.0]	0.89	0.375	111.0 [102.8, 119.0]	121.0 [104.5, 126.0]	1.96	0.050
**During Valsalva**								
BNd, mm	0.0 [−4.0, 8.0]	−5.0 [−12.0, 0.0]	4.19	<0.001	5.0 [−3.0, 9.0]	−9.0 [−14.0, −4.0]	4.79	<0.001
LAGAR, cm^2^	21.0 [17.0, 26.0]	23.0 [18.0, 28.8]	2.06	0.040	20.0 [16.0, 23.3]	23.0 [20.0, 29.0]	2.94	0.003
UTa (°)	−31.0 [−56.0, −11.0]	−46.0 [−63.8, −26.3]	2.37	0.018	−20.0 [−36.0, 0.3]	−61.0 [−83.0, −30.5]	4.17	<0.001
PVa (°)	150.09 (26.45)	159.84 (27.51)	2.55	0.013	145.70 (24.47)	167.04 (28.49)	3.46	0.001
Bladder distance, mm	0.0 [−5.0, 8.0]	−5.0 [−12.0, 0.0]	3.93	<0.001	5.0 [−3.0, 9.0]	−9.0 [−14.0, −4.0]	4.64	<0.001
URa (°)	56.39 (28.95)	64.89 (30.25)	2.02	0.046	47.99 (28.81)	77.41 (34.34)	3.98	<0.001
BNM, mm	29.0 [22.0, 35.0]	35.5 [29.0, 41.0]	4.29	<0.001	25.5 [19.8, 32.3]	36.0 [31.0, 43.5]	4.79	<0.001
Urethral funneling	24 (8.3)	17 (27.4)	18.08	<0.001	7 (9.2)	7 (25.9)	–	0.047

Continuous variables are mean (standard deviation) or median [interquartile range]; categorical variables are n (%). The Z/χ^2^/t column reports the value of the corresponding test statistic (χ^2^, t or Z, as appropriate); Fisher denotes Fisher’s exact test, which has no single statistic value. BNd, bladder-neck distance; LAGAR, levator ani hiatus area; UTa, urethral tilted angle; PVa, posterior vesicourethral angle; URa, urethral rotation angle; BNM, bladder-neck mobility. Fisher, Fisher’s exact test.

### Selection of predictors

Among the twelve candidate ultrasound parameters, LASSO (lambda.1se; coefficient paths and cross-validation in Figure 3) retained two: BNM and internal urethral orifice funneling. In the development cohort the ROC curve of continuous BNM had an AUC of 0.674 (95 % CI 0.593–0.754); the Youden index was maximal at 34.5 mm (sensitivity 54.8 %, specificity 74.7 %), and this development-cohort-derived threshold was used to define elevated BNM in both cohorts. Among clinical variables, univariable and multivariable logistic regression (Table 2) retained age (adjusted OR 1.130 per year, 95 % CI 1.046–1.221), vaginal delivery (4.786, 2.165–10.580) and overweight (2.177, 1.186–3.998); multiparity and neonatal birth weight were not independently significant.

**Figure 3:**
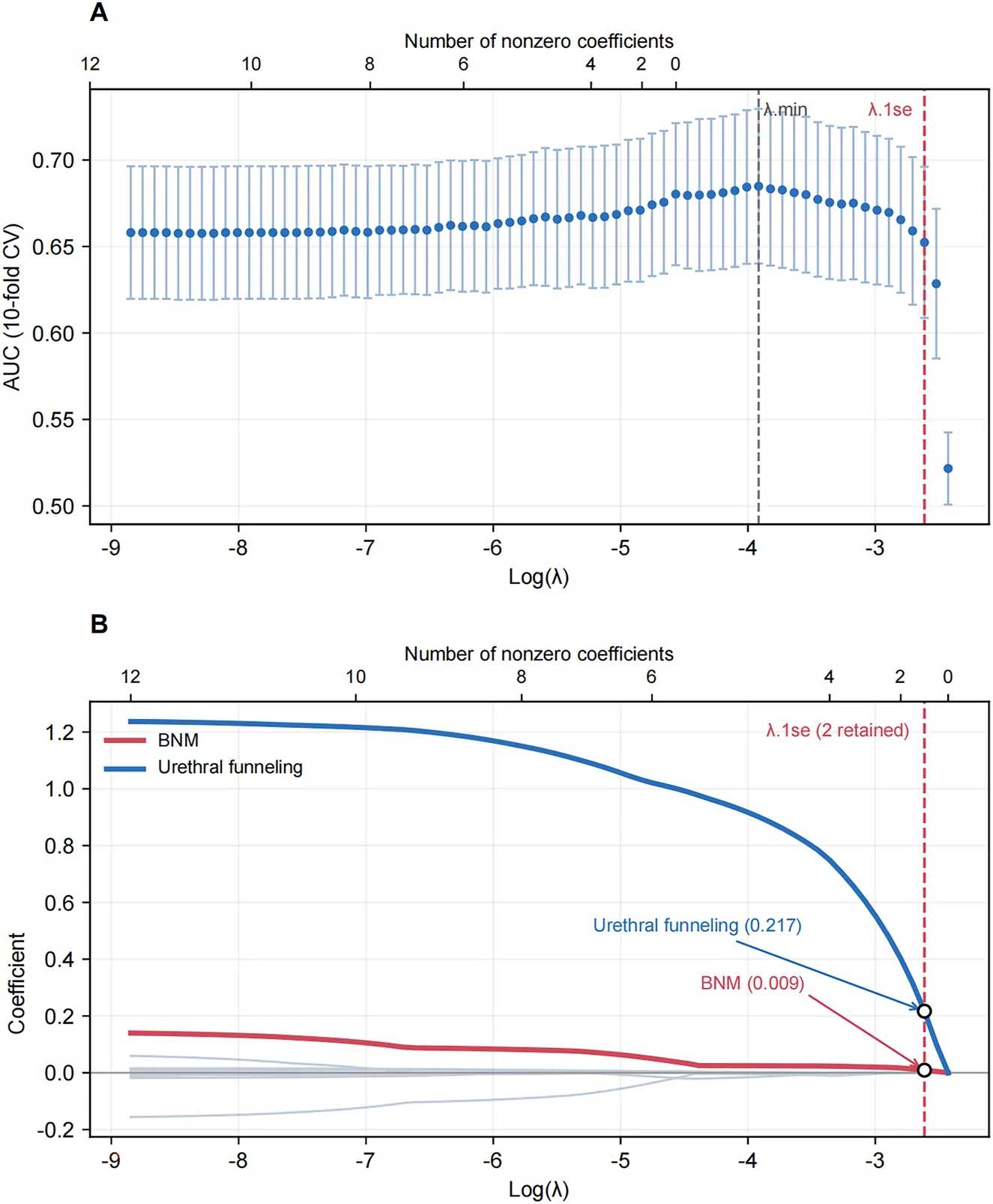
Least-absolute-shrinkage-and-selection-operator (LASSO) logistic regression on the twelve ultrasound parameters (development cohort). (A) Ten-fold cross-validated AUC against log(λ) (points, mean; whiskers, ±1 SE); the grey and red dashed lines mark λ.min (0.0199) and λ.1se (0.0733), respectively; the upper axis shows the number of nonzero coefficients. (B) LASSO coefficient paths; at λ.1se (red dashed line) two parameters – bladder-neck mobility and internal urethral orifice funneling – retained nonzero coefficients.

**Table 2:** Univariable and multivariable logistic regression of clinical characteristics for SUI in the development cohort.

Clinical variable	Univariable OR (95 % CI)	p-Value	Multivariable adjusted OR (95 % CI)	p-Value
Age, per year	1.121 (1.050–1.196)	0.001	1.130 (1.046–1.221)	0.002
Multiparity	2.074 (1.190–3.615)	0.010	1.477 (0.766–2.849)	0.244
Vaginal delivery	3.411 (1.618–7.193)	0.001	4.786 (2.165–10.580)	<0.001
Overweight (BMI>24)	2.267 (1.290–3.986)	0.004	2.177 (1.186–3.998)	0.012
Birth weight, per kg	0.994 (0.541–1.828)	0.985	1.126 (0.562–2.253)	0.738

### Final models

The full regression equations, coefficients and odds ratios are given in Table 3 to permit independent calculation. For the final combined Model 2, logit(p)=−7.054 + 0.130 × age (years) + 1.205 × vaginal delivery (1=yes) + 0.793 × overweight (1=BMI >24) + 0.761 × elevated BNM (1=>34.5 mm) + 1.301 × funneling (1=present).

**Table 3:** Full final logistic-regression models (development cohort): regression coefficients (β), odds ratios and coding, to permit independent calculation and validation.

Predictor (coding)	Model 1 β	Model 1 OR (95 % CI)	p-Value	Model 2 β	Model 2 OR (95 % CI)	p-Value
Intercept	−2.219	–	–	−7.054	–	–
Age (per year, continuous)	–	–	–	0.130	1.139 (1.061–1.222)	<0.001
Vaginal delivery (1=yes, 0=no)	–	–	–	1.205	3.337 (1.452–7.668)	0.004
Overweight (1=BMI>24, 0=no)	–	–	–	0.793	2.210 (1.186–4.121)	0.013
BNM elevated (1=>34.5 mm, 0=no)	1.194	3.301 (1.849–5.896)	<0.001	0.761	2.141 (1.143–4.010)	0.017
Urethral funneling (1=present)	1.292	3.641 (1.763–7.519)	<0.001	1.301	3.675 (1.719–7.856)	0.001

Model 1: logit(p)=−2.219 + 1.194 × [BNM>34.5]+1.292 × [funneling]. Model 2 equation is given in the text. OR, odds ratio; CI, confidence interval.

### Discrimination, calibration and clinical utility

Discrimination for both models in both cohorts is summarised in Table 4 and Figure 4. In the development cohort, Model 2 achieved a significantly higher C-statistic than Model 1 (0.780 [0.717–0.843] vs. 0.703 [0.634–0.771]; paired DeLong Z=3.053, p=0.002); the 2,000-resample bootstrap-corrected C-statistic of Model 2 was 0.763 (apparent optimism 0.017). In the temporal test cohort the C-statistics were 0.805 (0.716–0.894) for Model 2 and 0.737 (0.633–0.841) for Model 1 (paired DeLong Z=1.474, p=0.141). The slightly higher point estimate in the test cohort should be interpreted cautiously because the test cohort contained only 27 events and the confidence intervals of the two cohorts overlap. The Model 2 nomogram for bedside calculation is shown in Figure 5. Calibration of Model 2 was acceptable in both cohorts (development: intercept 0.000, slope 1.000, Brier 0.121; test: intercept 0.704, slope 1.054, Brier 0.161; Hosmer–Lemeshow p=0.926 and 0.168; Figure 6); Model 1 showed poorer calibration in the test cohort (Hosmer–Lemeshow p=0.012). Decision-curve analysis suggested that Model 2 offered potential net benefit over treat-all and treat-none at selected low-to-moderate, clinically relevant thresholds in both cohorts, and did so more consistently than Model 1 (Figure 7); this should not be read as continuous benefit across the entire threshold range. As with discrimination, the temporal-test decision curve rests on only 27 outcome events and should therefore be regarded as indicative rather than definitive.

**Table 4:** Discrimination and calibration of Model 1 and Model 2 in both cohorts (C-statistic with DeLong 95 % CI).

Cohort	Model	C-statistic (95 % CI)	Calibration intercept	Calibration slope	Brier score	Hosmer–Lemeshow P	P, Model 2 vs. Model 1 (paired DeLong)
Development (n=351)	Model 1 (ultrasound)	0.703 (0.634–0.771)	0.000	1.000	0.132	0.243	0.002
Development (n=351)	Model 2 (combined)	0.780 (0.717–0.843)	0.000	1.000	0.121	0.926	
Temporal test (n=103)	Model 1 (ultrasound)	0.737 (0.633–0.841)	1.245	1.435	0.169	0.012	0.141
Temporal test (n=103)	Model 2 (combined)	0.805 (0.716–0.894)	0.704	1.054	0.161	0.168	

Bootstrap (2000 resamples) optimism-corrected C-statistic of Model 2 in the development cohort=0.763 (apparent optimism 0.017).

**Figure 4:**
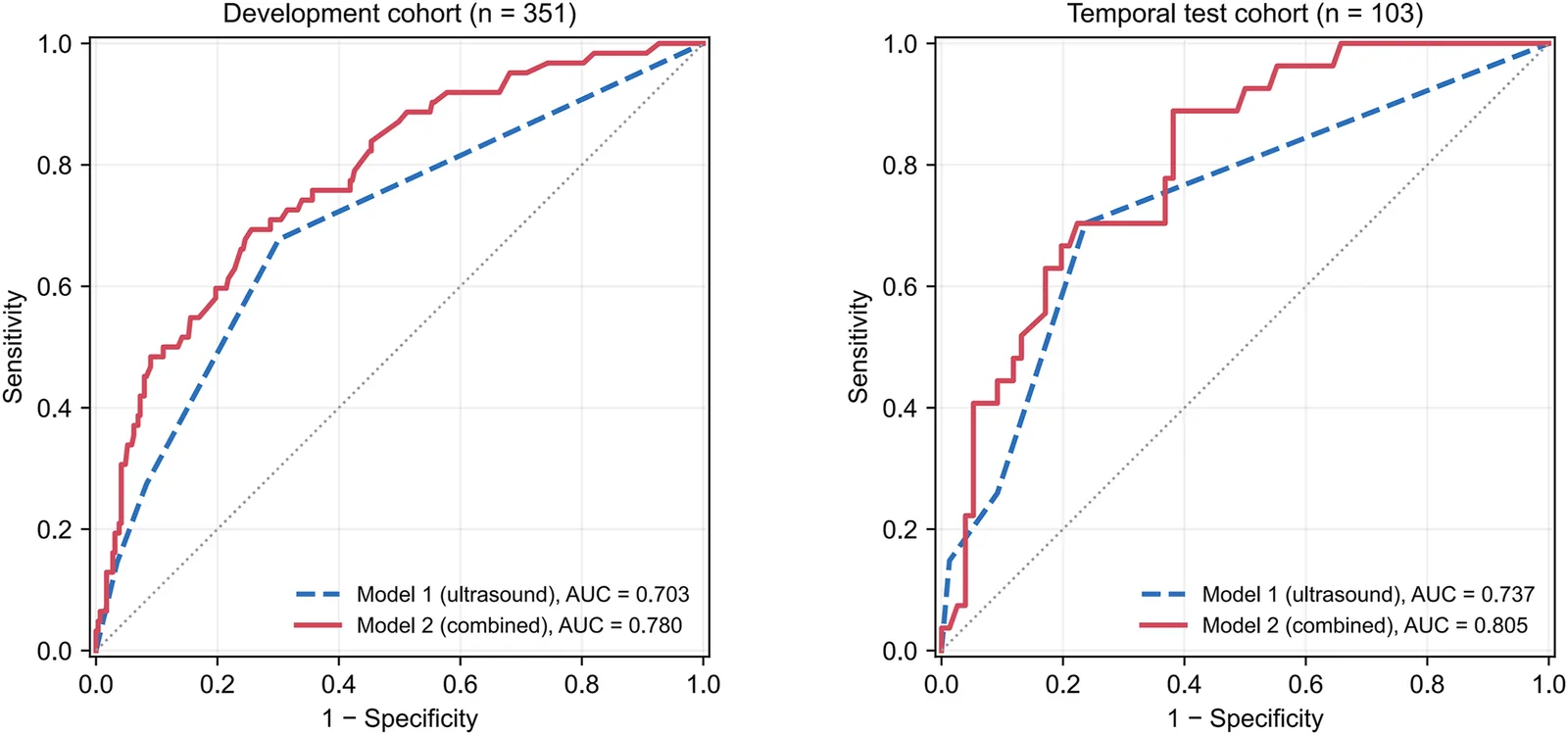
Receiver-operating-characteristic curves of Model 1 and Model 2 in the development and temporal test cohorts, with DeLong 95 % confidence intervals (Model 1: 0.703 and 0.737; Model 2: 0.780 and 0.805).

**Figure 5:**
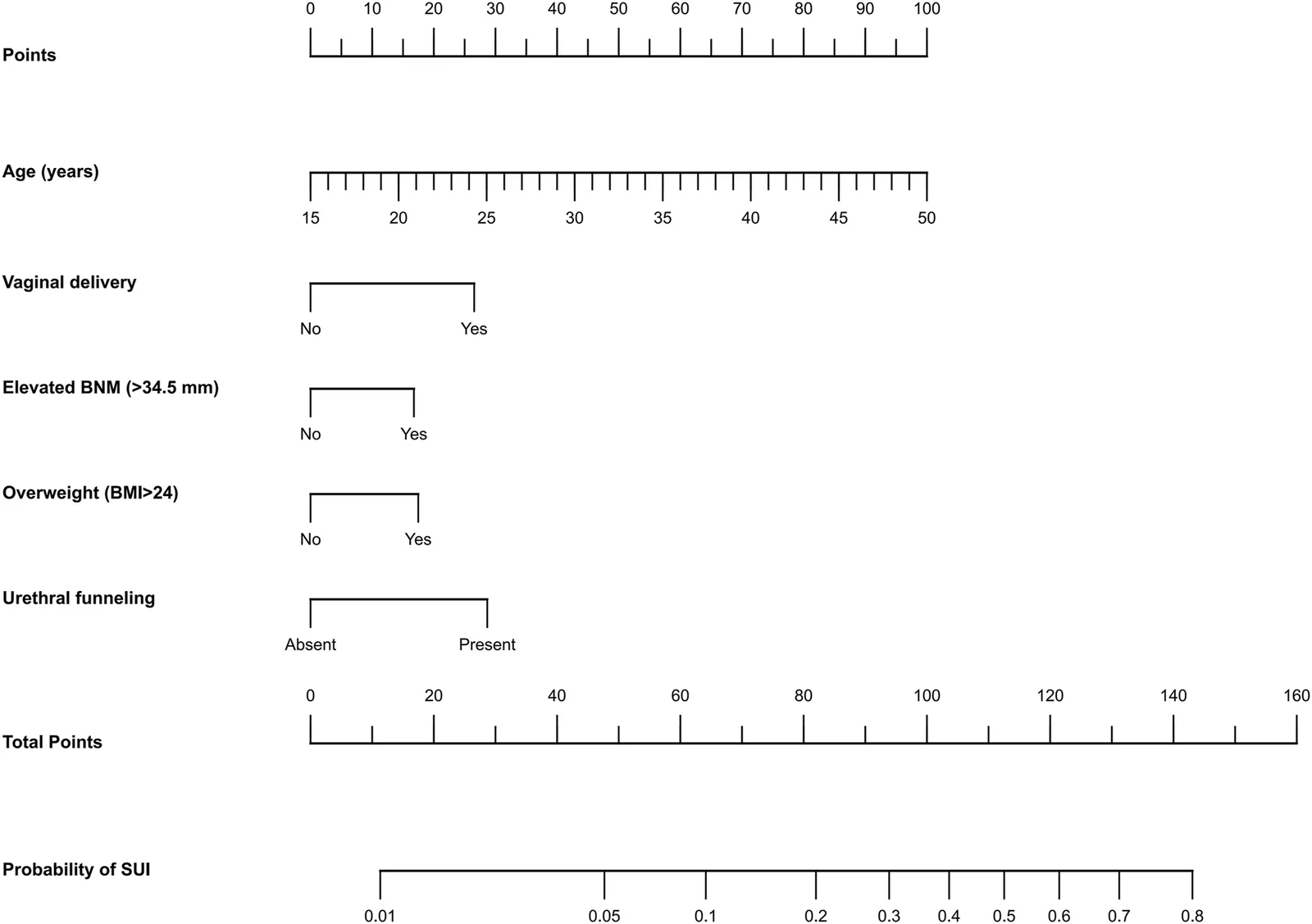
Nomogram of the combined Model 2. Points for age, mode of delivery, overweight, elevated BNM and funneling are summed on the total-points axis and converted to the probability of identifying SUI at six weeks postpartum.

**Figure 6:**
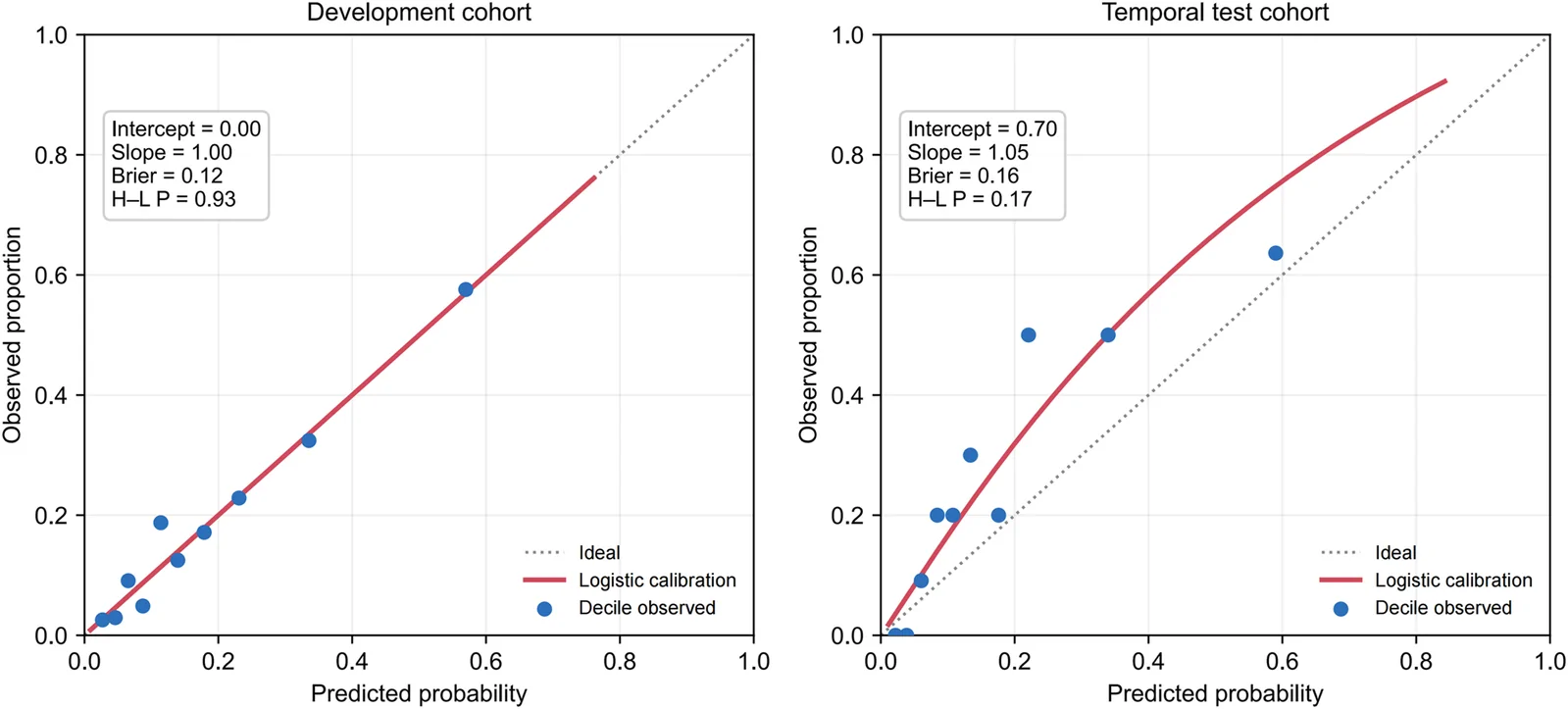
Calibration plots of Model 2 in the development and temporal test cohorts, with calibration intercept, slope, Brier score and Hosmer–Lemeshow p annotated; the diagonal denotes perfect calibration.

**Figure 7:**
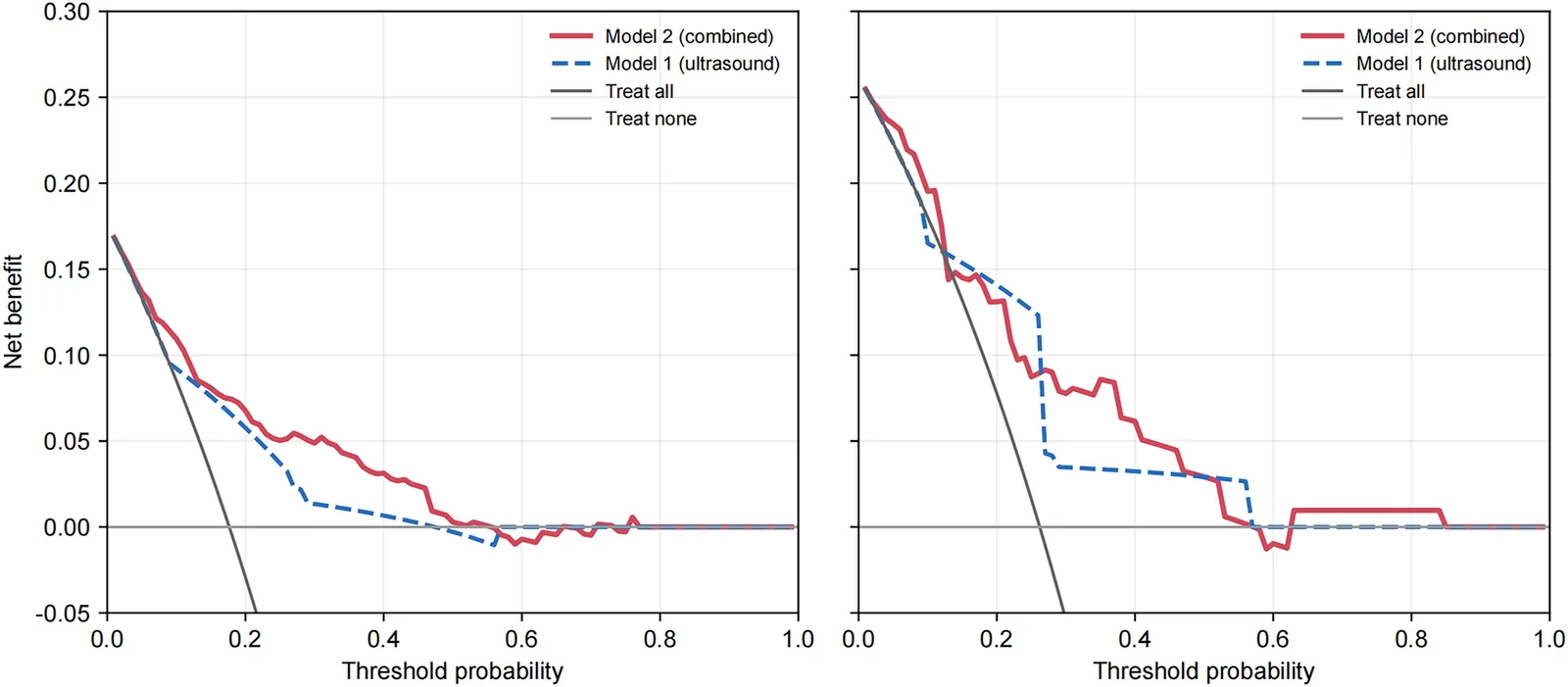
Decision-curve analysis for Model 1, Model 2, treat-all and treat-none in (A) the development cohort (n=351) and (B) the temporal test cohort (n=103). Model 2 showed potential net benefit over a wider threshold-probability range than Model 1 and the two reference strategies, although the temporal-test estimates rest on only 27 outcome events and should be interpreted as indicative pending external validation.

### Sensitivity analysis with continuous predictors

When BMI and BNM were retained as continuous variables, BNM remained independently associated with SUI (OR 1.037 per mm, 95 % CI 1.002–1.073; p=0.041), whereas continuous BMI was not significant (OR 1.044 per kg/m^2^, 0.941–1.158; p=0.415). Discrimination was essentially unchanged (development C-statistic 0.758; test 0.790) and did not differ significantly from the dichotomised Model 2 in either cohort (paired DeLong p=0.123 and 0.517), supporting the stability of the main model to variable coding; the continuous analysis is presented to acknowledge the information loss from categorisation. Full coefficients of this continuous model and its discrimination are provided in Supplementary Table S2.

## Discussion

In this retrospective single-centre study of 454 women reviewed at the routine six-week postpartum visit, a five-variable model combining three readily available clinical characteristics (age, vaginal delivery and overweight) with two transperineal ultrasound findings (BNM >34.5 mm and internal urethral orifice funneling) identified women with concurrent SUI with a C-statistic of 0.780 in the development cohort and 0.805 in a temporally separated internal-validation cohort, a calibration slope near unity (with an intercept shift reflecting the period-to-period rise in SUI prevalence) and net benefit at selected low-to-moderate thresholds on DCA. Adding the three clinical characteristics to the ultrasound variables significantly improved discrimination in the development cohort (Model 2 vs. Model 1). We emphasise that, because predictors and outcome were recorded at the same visit, the model identifies prevalent SUI (case-finding) rather than predicting future, incident SUI.

### Mechanistic interpretation of bladder-neck mobility

BNM was the strongest ultrasound discriminator, consistent with prior work [15], 16], 18], 19]. Mechanistically, pregnancy and vaginal delivery stretch and may partially denervate the levator ani, the pubourethral ligaments and the connective-tissue scaffolding of the bladder neck [5], 13], 34]. When this supportive hammock is compromised, rises in intra-abdominal pressure are transmitted equally to the bladder and a poorly supported proximal urethra; the bladder neck becomes hypermobile, descends and rotates during Valsalva, and the internal urethral orifice may funnel open, defeating urethral closure and producing leakage on exertion [14], 17], 20]. BNM and funneling should therefore be understood not as isolated imaging curiosities but as visible consequences of impaired pelvic-floor support – an interpretation supported by the postpartum recovery trajectory of these measurements [6].

### Comparison with previous models

Existing SUI prediction models rely predominantly on questionnaires and obstetric history [24], 25]; the recent rural-Fujian model, for example, used only clinical variables [25], while a 2024 scoping review documented the rapid but heterogeneous growth of machine-learning approaches in female urinary incontinence [26]. Chen et al. combined ultrasound with clinical factors during pregnancy [24], whereas our population is assessed at six weeks after delivery, when conservative rehabilitation is conventionally initiated. Our paired comparison (Model 2 vs. Model 1) provides within-cohort evidence that adding clinical characteristics to the two ultrasound variables improves discrimination (paired DeLong p=0.002 in the development cohort; the smaller test cohort showed the same direction but did not reach significance, p=0.141). We did not, however, fit a clinical-only reference model, so the incremental value of ultrasound over clinical factors alone cannot be quantified directly; this comparison is an important next step. The continuous-variable sensitivity analysis showed that the ultrasound contribution did not hinge on the chosen BNM cut-off.

### Choice of ultrasound route and feasibility for screening

Pelvic-floor ultrasound has evolved substantially over two decades [13], 23], and bladder-neck motion is not unique to the 4D transperineal route: transabdominal ultrasound can also quantify bladder-neck movement and pelvic-floor muscle contraction and may be cheaper, more acceptable and easier to repeat [22]. We acknowledge that 4D transperineal ultrasound requires specialised equipment and an experienced operator and is therefore not feasible for universal postpartum screening in every setting. The present five-variable model should be viewed as a specialist-clinic tool; future work should test whether simplified two-dimensional or transabdominal protocols that measure only BNM and funneling preserve discrimination in primary care, and whether functional imaging of muscle contraction, dynamic bladder-neck behaviour and response to rehabilitation can add prognostic information [6], 21].

### Linking identification to intervention

Identification is only meaningful if it changes care. Under IUGA/ICS conservative-management terminology [9], a woman flagged as high probability at six weeks can be offered supervised PFMT, physiotherapy, behavioural/lifestyle advice and a structured postpartum rehabilitation programme; Cochrane evidence supports PFMT as effective for urinary incontinence in women [10], and early postpartum exercise favourably influences pelvic-floor muscle function [11], 12]. Operationally, a positive result at a low-to-moderate threshold would trigger further assessment and referral for conservative management (supervised PFMT/physiotherapy), not treatment or surgery; the operating cut-off should be chosen after weighing the low burden and low risk of PFMT against local capacity. DCA estimates potential net benefit under such assumed decisions; it does not by itself prove that patients benefit, which would require an impact study [33], 35].

### Interpretation of performance

The nomogram is deliberately parsimonious [32]. Its calibration slope remained close to unity in the temporal test cohort (1.054), indicating an appropriate spread of predicted risks with little evidence of overfitting, whereas the calibration intercept of 0.704 reflects systematic under-prediction consistent with the higher SUI prevalence in the later recruitment period (26.2 % vs. 17.7 %); this temporal baseline shift supports recalibration-in-the-large at deployment and, together with the small validation sample, underscores the need for prospective external validation. Nevertheless, the test-cohort point estimate (0.805) was numerically higher than the development estimate despite a smaller sample, and it rests on only 27 events; the overlapping confidence intervals and the bootstrap optimism correction (0.763) are more realistic indicators of expected performance. We therefore describe discrimination as moderate-to-good rather than “excellent” and regard the model as preliminary.

### Limitations

Several limitations merit consideration. First, this retrospective single-centre study used a temporal internal-validation cohort with only 27 SUI events. The validation estimates were therefore imprecise, and the numerical advantage of Model 2 over Model 1 did not reach statistical significance (paired DeLong p=0.141). Prospective multicentre external validation is required. Second, predictors and symptom-defined SUI were assessed at the same visit; the model therefore supports concurrent case-finding rather than prognosis, and outcome misclassification remains possible without objective confirmation. Third, BMI and BNM were dichotomised. Although the continuous-predictor sensitivity analysis yielded similar discrimination, the 34.5-mm BNM threshold requires external confirmation. Fourth, all scans were obtained by a single operator without formal reproducibility assessment, and several obstetric variables, including operative vaginal delivery and episiotomy, were unavailable. Finally, without a clinical-only comparator, the incremental value of ultrasound beyond routine clinical characteristics remains undetermined.

### Conclusions

A clinical–transperineal ultrasound nomogram incorporating age, vaginal delivery, overweight, BNM and urethral funneling can help identify women with SUI at the six-week postpartum visit and guide early conservative management; the combined model outperformed an ultrasound-only model. The findings are preliminary and should be confirmed by prospective external validation and by an evaluation of the model’s impact on clinical decision-making and patient outcomes before routine implementation.

## Supplementary Material

Supplementary Material
