# Supplementary material for: An ultrasound model for stress urinary incontinence at six weeks postpartum
Source: Open Med (Wars). 2026 Oct 30;21(1):20261549. doi: 10.1515/med-2026-1549 (PMC13718988; doi:10.1515/med-2026-1549)
Supplement: Supplementary file 1 [file j_med-2026-1549_suppl_001.docx]

**Supplementary Table S1. Overall characteristics of the development and temporal test cohorts.**

| **Characteristic** | **Development cohort (n = 351)** | **Temporal test cohort (n = 103)** | Z/χ²/ t | ***P*** |
| --- | --- | --- | --- | --- |
| Age (years) | 29.0 [26.0, 31.0] | 29.0 [26.5, 32.0] | 1.06 | 0.291 |
| Multiparity | 125 (35.6%) | 38 (36.9%) | 0.06 | 0.812 |
| Vaginal delivery | 236 (67.2%) | 55 (53.4%) | 6.63 | 0.010 |
| BMI (kg/m²) | 22.4 [20.2, 24.2] | 22.5 [20.6, 24.5] | 0.73 | 0.463 |
| Overweight (BMI > 24 kg/m²) | 105 (29.9%) | 39 (37.9%) | 2.32 | 0.127 |
| Birth weight (kg) | 3.3 [3.0, 3.6] | 3.2 [3.1, 3.5] | 0.93 | 0.355 |
| BNd at rest (mm) | 29.12 (3.21) | 28.75 (2.85) | 1.12 | 0.263 |
| LAGAR at rest (cm²) | 14.0 [12.0, 16.0] | 13.0 [12.0, 15.0] | 1.97 | 0.049 |
| UTa at rest (°) | 24.0 [11.0, 36.0] | 26.0 [13.5, 38.0] | 1.21 | 0.225 |
| PVa at rest (°) | 115.0 [104.0, 127.0] | 113.0 [103.0, 122.5] | 1.48 | 0.138 |
| BNd during Valsalva (mm) | −1.0 [−6.0, 7.0] | 2.0 [−6.0, 8.0] | 0.76 | 0.449 |
| LAGAR during Valsalva (cm²) | 21.0 [17.5, 26.0] | 21.0 [17.0, 25.5] | 0.92 | 0.359 |
| UTa during Valsalva (°) | −34.0 [−58.0, −12.0] | −29.0 [−56.5, −5.5] | 1.28 | 0.202 |
| PVa during Valsalva (°) | 151.81 (26.86) | 151.29 (27.13) | 0.17 | 0.863 |
| Bladder distance during Valsalva (mm) | −2.0 [−6.0, 7.0] | 2.0 [−6.0, 8.0] | 0.83 | 0.406 |
| URa during Valsalva (°) | 57.89 (29.32) | 55.70 (32.87) | 0.61 | 0.542 |
| BNM (mm) | 30.0 [22.0, 36.0] | 29.0 [22.0, 35.5] | 0.79 | 0.429 |
| Urethral funneling | 41 (11.7%) | 14 (13.6%) | 0.27 | 0.601 |
| SUI identified | 62 (17.7%) | 27 (26.2%) | 3.69 | 0.055 |

Data are presented as mean (SD), median [IQR], or n (%), as appropriate. *P* values were calculated using Student’s t test, the Mann–Whitney U test, or the χ² test, as appropriate. BMI, body mass index; BNd, bladder-neck distance; BNM, bladder-neck mobility; IQR, interquartile range; LAGAR, levator ani hiatus area; PVa, posterior vesicourethral angle; SD, standard deviation; SUI, stress urinary incontinence; URa, urethral rotation angle; UTa, urethral tilt angle.

**Supplementary Table S2. Sensitivity analysis retaining body mass index and bladder-neck mobility as continuous predictors.**

**A.** Multivariable logistic regression of the continuous-predictor model.

| **Predictor** | **β** | **Adjusted OR (95% CI)** | ***P*** |
| --- | --- | --- | --- |
| Age (per year) | 0.131 | 1.140 (1.062–1.225) | <0.001 |
| Vaginal delivery (1 = yes) | 1.168 | 3.217 (1.386–7.467) | 0.007 |
| BMI (per kg/m²) | 0.043 | 1.044 (0.941–1.158) | 0.415 |
| BNM (per mm) | 0.036 | 1.037 (1.002–1.073) | 0.041 |
| Urethral funneling | 1.216 | 3.373 (1.586–7.174) | 0.002 |
| Intercept | −8.574 | — | — |

Odds ratios for age and bladder-neck mobility are reported per year and per millimetre, respectively; the odds ratio for body mass index is reported per kg/m².β, regression coefficient; BMI, body mass index; BNM, bladder-neck mobility; CI, confidence interval; OR, odds ratio.

**B.** Discrimination of the continuous-predictor model versus the dichotomised main Model 2, with paired DeLong comparisons in each cohort.

| **Model** | **Development C-statistic (95% CI)** | **Temporal test C-statistic (95% CI)** |
| --- | --- | --- |
| Main Model 2 (BMI > 24 kg/m² and BNM > 34.5 mm, dichotomised) | 0.780 (0.717–0.843) | 0.805 (0.716–0.894) |
| Continuous-predictor sensitivity model | 0.758 (0.691–0.826) | 0.790 (0.695–0.885) |
| Paired DeLong P (continuous vs main) | 0.123 | 0.517 |

*Note.* Continuous body mass index was not independently associated with SUI, and discrimination did not differ significantly from that of the main model in either cohort. These findings support the robustness of the main results to the coding of body mass index and bladder-neck mobility.
